# British Ornithology

**Published:** 1811-03

**Authors:** 


### BRITISH ORNITHOLOGY.

Two numbers of the work on British Ornithology, by Mr. George Graves, of which we gave notice in our number for January, have appeared; as this work promises to be complete, and contains much information interesting to the naturalist, we conceive a monthly report of the contents of each number will be acceptable to most of our readers. Mr. Graves has in the preface given the outline of the plan he intends to pursue. We notice in the specific characters he differs from the usual method in not naming the colours, the correctly coloured plates accompanying superseding this necessity. We also observe, that the plates and letter-press are not numbered, which is a judicious omission, as it gives an opportunity of arranging the subjects when the work may be completed, according to their place in the system. Number I. contains a very good figure of Loxia Curvirostra, the *Cross-bill*; this beautiful species is very highly coloured; though it is observed to vary in colour, we do not recollect having seen one so richly marked. Avocetta Recurvirostra, the *Avocet*; this beautiful species is not very common in this country, being only met with on the sea shore or in the fenny districts. Scolopax Arquata, the *Common Curlew*, a very correct figure. Motacilla Regulus, the *Golden-crested Wren*, a beautiful figure, it is observed that this is the smallest species of European bird. Parus Cærulus, the *Blue Titmouse*, a masterly drawing, but we doubt whether the colours in this instance are not too dull. Alcedo Ispida, the *Kingfisher*, a good figure and very beautifully coloured, the description of this bird is particularly interesting. Number 2, contains a fine figure with a very pleasing history of the Tetrao Perdix, the common *Partridge*. Anas Tadorna, the *Shieldsake*, a very correct and picturesque figure, Hematopus Ostralegus, the *Pied Oyster-catcher*, Corvus Cornix, the *Hooded Crow*: in a quotation from Pennant we are informed this is the only species of crow found in the Scottish Isles. Upupa Epops, the *Hoopoe*, a very rare migrative species. This drawing and engraving were executed for (Mr. Graves's deceased relative) William Curtis, author of the Flora Londinensis. Ardea Major, the *Common Heron*, this is one of the most elegant figures we remember to have seen, the description is good, and the history contains much interesting and amusing information illustrative of the habits and manners of this beautiful species.

METEOROLO-

